# Genetic Variants in Circadian Rhythm Genes and Self-Reported Sleep Quality in Women with Breast Cancer

**DOI:** 10.5334/jcr.184

**Published:** 2019-07-01

**Authors:** Tricia D. LeVan, Peng Xiao, Gaurav Kumar, Kevin Kupzyk, Fang Qiu, David Klinkebiel, James Eudy, Kenneth Cowan, Ann M. Berger

**Affiliations:** 1 University of Nebraska Medical Center, US

**Keywords:** sleep deficiency, sleep-wake disturbance, sleep quality, circadian genes, genetic variation

## Abstract

**Introduction:**

Women diagnosed with breast cancer (BC) are at increased risk of sleep deficiency. Approximately 30–60% of these women report poor sleep during and following surgery, chemotherapy, radiation therapy, and anti-estrogen therapy. The purpose of this study was to examine the relationship between genetic variation in circadian rhythm genes and self-reported sleep quality in women with BC.

**Methods:**

This cross-sectional study recruited women with a first diagnosis of breast cancer at five sites in Nebraska and South Dakota. Sixty women were included in the study. Twenty-six circadian genes were selected for exome sequencing using the Nextera Rapid Capture Expanded Exome kit. 414 variants had a minor allele frequency of ≥5% and were included in the exploratory analysis. The association between Pittsburgh Sleep Quality Index (PSQI) score and genetic variants was determined by two-sample t-test or ANOVA.

**Results:**

Twenty-five variants were associated with the PSQI score at p < 0.10, of which 19 were significant at p<0.05, although the associations did not reach statistical significance after adjustment for multiple comparisons. Variants associated with PSQI were from genes CSNK1D & E, SKP1, BHLHE40 & 41, NPAS2, ARNTL, MYRIP, KLHL30, TIMELESS, FBXL3, CUL1, PER1&2, RORB. Two genetic variants were synonymous or missense variants in the BHLHE40 and TIMELESS genes, respectively.

**Conclusions:**

These exploratory results demonstrate an association of genetic variants in circadian rhythm pathways with self-reported sleep in women with BC. Testing this association is warranted in a larger replication population.

## Introduction

Breast cancer remains the second leading cause of cancer deaths among women [1]. While overall survivorship has increased over time, sleep deficiency is one of the most frequent and distressing symptoms reported by women with breast cancer and has a negative impact on quality of life and functional status [2,3]. About 30–60% of women with breast cancer report problems sleeping at diagnosis and the percent increases during chemotherapy treatments [4,5]. One of the main adverse events from aromatase inhibitors that lead to drug discontinuance is sleep disorders [6]. Several predictors of sleep deficiency have been identified but mechanisms responsible for poor sleep in patients with cancer are poorly understood [7,8].

Significant heritability of sleepiness, usual bedtime, and usual sleep duration has been discovered [9], which suggests that genetic factors may make some individuals more susceptible to sleep disturbance. A series of publications detail associations between cytokine gene variations and self-reported sleep or symptom clusters that included sleep in patients with cancer [10,11,12,13,14]. Also, evidence suggests cytokine dysregulation is associated with sleep disturbance in humans [15].

Circadian clocks synchronize physiological and behavioral rhythms with time. Dysregulated expression of circadian clock-related genes is greatly affected by polymorphic variants and has been associated with cancer [16]. An interesting report by Truong and team [17] examined breast cancer risk, night work, and circadian clock gene polymorphisms. The team examined polymorphisms from 577 validated single nucleotide polymorphisms (SNPs) in 23 circadian clock genes in a large sample of breast cancer cases and controls. Two SNPs in retinoic acid receptor-related orphan receptor (RORA; rs1482057 and rs12914272) were associated with breast cancer in the whole sample and among post, but not pre-menopausal women. Authors summarize that the results support the hypothesis that circadian clock gene variants modulate breast cancer risk.

Little attention, however, has focused on genetic associations between circadian clock genes and sleep deficiency in patients with cancer. Two systematic reviews [18,19] summarize genomic variants associated with cancer-related fatigue but no circadian clock genes are included.

Based on this knowledge, the purpose of this exploratory study was to analyze correlations between self-reported sleep index values of sleep quality and genetic variants in 26 circadian clock genes in women with breast cancer.

## Methods

### Design

A cross-sectional feasibility study design was used. The parent study examined data from the Breast Cancer Collaborative Registry (BCCR) questionnaire to understand risk factors predicting sleep quality in patients with breast cancer [20].

### Study population

The BCCR was used to locate cases collected by UNMC/Nebraska Medicine, Omaha, NE from January 2008 to January 2017. Inclusion criteria in the parent study were: 1) women with a first breast cancer diagnosis; and 2) at any phase of the cancer trajectory. Additional inclusion criteria for this exploratory study included: 3) completed the Pittsburgh Sleep Quality Index (PSQI) in the BCCR questionnaire and 4) had a blood sample that had been analyzed using exome sequencing. Exclusion criteria were those: 1) diagnosed with recurrent breast cancer, and 2) males. The Institutional Review Board (IRB) of the University of Nebraska Medical Center approved the study. At enrollment, patients provided informed consent for use of the data in clinical studies. Women were invited to participate during routine oncology appointments.

### Breast Cancer Collaborative Registry (BCCR) Questionnaire

The BCCR, which is a part of the integrated Cancer Repository for Cancer Research (iCaRe2), was developed in collaboration with breast cancer experts and research questions were standardized to satisfy the needs of all the centers [21]. The questionnaire contains standard data to provide a comprehensive review of the patient’s demographic, medical, tumor, lifestyle, environmental, quality of life, and sleep quality that could influence breast cancer diagnosis and survivorship. Demographic data include variables such as participant’s age, race/ethnicity, marital status, and educational status. Medical data include height/weight/BMI and a list of chronic conditions but no comorbidity index; gynecologic data such as menstrual status, pregnancy, breast-feeding, and birth control; and breast cancer data such as therapies received, functional changes, and symptoms since surgery or completing therapy. Tumor data include stage and receptor status. Lifestyle data include history of smoking, alcohol consumption, and physical activity. Environmental factors include annual household income and history of night or rotating shiftwork. Measures of physical and mental health status and subjective sleep quality complete the questionnaire. More information about the BCCR is published [20]. All participants completed the BCCR questionnaire either at a clinic appointment or at home and returned it by United States Postal Service.

### Sleep

Subjective sleep quality during the past month was measured using the 19-item Pittsburgh Sleep Quality Index (PSQI) [22,23]. A global score and seven component scores were obtained, including sleep quality, sleep latency, sleep duration, habitual sleep efficiency, sleep disturbances, sleeping medication use, and daytime dysfunction. Components are scored on a 0–3 scale and combined with equal weights, yielding a global score (0–21). Higher scores indicate more severe complaints and poor sleep quality. Cronbach’s alpha for the global PSQI was reported as 0.80 and was 0.71 in this study. A global PSQI score >5 has a sensitivity of 89.6% and a specificity of 86.5% in identifying poor sleepers. Optional questions 10–11 were not included.

### Genetic Analysis

Genomic DNA was isolated from blood and sequenced on n = 128 participants from the parent study. Twenty-six circadian genes were selected for analysis based on results from the 2008 Sleep Research Society Presidential Task Force on Sleep/Circadian Rhythm SNP Gene Array Initiative and the report by Troung [17]. Exome sequencing was performed using the Nextera Rapid Capture Expanded Exome kit (Illumina, San Diego, CA). Target DNA included exons, untranslated regions (UTRs) and miRNAs. Following the manufacturers’ suggested protocol, 50 ng of genomic DNA from each sample was subjected to “tagmentation” to generate a genome wide library of fragments. The targeted content was captured by hybridization of the library to the oligonucleotides provided by the manufacturer. The resultant exome library for each sample was quantified by qPCR and 10 pM of the pooled libraries were loaded three samples per lane on an Illumina HiSeq2500 DNA sequencer and 150 bp paired-end runs were performed.

### Bioinformatic Methods

We used an established variant calling pipeline using bcbio-nextgen python toolkit (https://github.com/bcbio/bcbio-nextgen) for the exome sequencing data. Initially, raw sequencing reads in FASTQ format were trimmed by the fqtrim tool.

(https://ccb.jhu.edu/software/fqtrim) to remove adapters, terminal unknown bases (Ns) and low quality 3’ regions (Phred score <30). The quality of trimmed sequence reads were assessed using quality control tool FastQC [24]. The trimmed reads passing FastQC were aligned to the hg19 reference genome with Borrows-Wheeler Aligner [25] and further processed through the GATK pipeline [26,27] for base quality score recalibration, INDEL realignment, and mark duplicates, according to GATK’s best practices recommendations [27,28]. Four variant callers, MuTect [29], freebayes [30], VarDict [31], and VarScan [32] were used to call variants from the sequencing data. All the called germline variants from the 128 blood samples were saved into 128 Variant Call Format (VCF) files. We further wrote a perl script to extract variants within the range of the 26 candidate genes (with 1Kb flanking) from the 128 germline VCF files and a python script to format the extracted variants into an excel table for follow-up association analyses.

### Data Analysis

Due to positive skew, the primary outcome of sleep index value (PSQI) was log transformed to meet normality assumptions. Genetic variants with a minor allele frequency (MAF) less than 5% were excluded in the analysis. For each genetic variant, the association between log-transformed sleep index value (PSQI) and the genetic variant was determined by two-sample t-test or ANOVA. SAS software version 9.4 (SAS Institute Inc., Cary, NC) was used for all analyses. Linkage disequilibrium was determined using Haploview software [33].

## Results

### Demographic and Clinical Characteristics of Participants

Participants’ baseline demographic and clinical characteristics were representative of the breast cancer population (Table 1). Women’s mean age was 58.6 (SD = 13.6; range 27–85; median 59.6) years and they were predominantly Non-Hispanic whites (88.3%); married (62.7%); had some post-secondary education (74.1%); and were diagnosed at Stage I or Stage II breast cancer (81.4%).

**Table 1:** Study population characteristics, n = 60.

Variable	Categories

Age, mean ± SD	58.6 ± 13.6
Ethnicity	
White	53 (88.3%)
Non-White	7 (11.7%)
Hispanic	
Yes	1 (1.9%)
No	53 (89.1%)
Marital Status*	
Partnered	37 (62.7%)
Non-Partnered	22 (37.3%)
Education*	
≤ High school	14 (25.9%)
Some College	19 (35.2%)
≥ College	21 (38.9%)
Income*	
< 25k	13 (23.2%)
25k to 75k	22 (39.3%)
> 75k	21 (37.5%)
Ever Worked Night Shift*	
Yes	11 (4.0%)
No	14 (56.0%)
Alcohol Drinks/Week*	
<1/week	22 (51.2%)
1 day/week	7 (16.3%)
2–3 days/week	8 (18.6%)
4–5 days/week	4 (9.3%)
6–7 days/week	2 (4.7%)
Current Smoker*	
Yes	5 (27.8%)
No	13 (72.2%)
Stage of Breast Cancer*	
I	24 (40.7)
II	24 (40.7)
III	10 (16.9)
IV	1 (1.7)

* missing data.

### Selection of Genetic Variants

**Figure 1:**
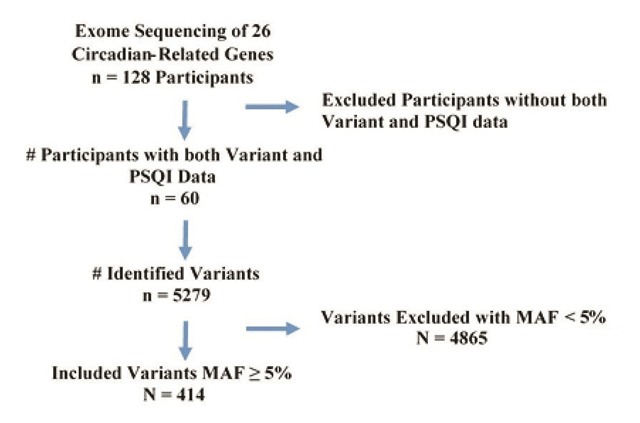
STROBE (Strengthening the Reporting of Observational studies in Epidemiology) diagram. Individuals were excluded from analysis for missing PSQI and genetic variant data and a MAF < 5%.

Sequencing data from 26 circadian rhythm genes were obtained from 128 subjects; however, only 60 subjects had both sequencing data and self-reported PSQI scores. For these 60 subjects, we identified 5,279 genetic variants, of which 4,865 were excluded in the analysis because of a minor allele frequency (MAF) less than 5%. The remaining 414 variants were analyzed for their association with PSQI scores (continuous variable). Figure 1 illustrates the STROBE (Strengthening the Reporting of Observational studies in Epidemiology) diagram and the final sample for analysis.

### Association between Genetic Variants and PSQI Score

Tables 2 and 3 list 25 genetic variants that were associated with the global PSQI score at p < 0.10, and 19 of these were significant at p < 0.05. The associations did not meet statistical significance after adjustment for multiple comparisons, possibly because of the exploratory nature of the study (large number of comparisons with a small samples). These genetic variants were found throughout the genome (chromosomes 2, 3, 5, 7, 9, 11, 12, 13, 22) and represented 15 genes including CSNK1D & E, SKP1, BHLHE40 & 41, NPAS2, ARNTL, MYRIP, KLHL30, TIMELESS, FBXL3, CUL1, PER1 & 2, and RORB. Most variants were found in intronic and untranslated regions except for two, which were synonymous and missense variants in BHLHE40 and TIMELESS genes, respectively. Mean log-transformed PSQI scores were higher for 10 polymorphisms among heterozygous subjects, relative to those with the homozygous genotype, the remaining variants were lower. Linkage disequilibrium was determined only on chromosome 3 (rs908078 vs rs34870629, rs34883305, rs74439275; r^2^ = 0.52) and chromosome 5 (rs2110585 vs rs3815506, rs73791514; r^2^ = 0.85).

**Table 2:** Top 25 genetic variants associated with PSQI (p-value < 0.1).

ID	Chromosome #	Chromosome Location	Gene Name	Variant Annotation

rs3841571	chr2	101582245	NPAS2	Deletion/Insertion
rs1053095	chr2	101612584	NPAS2	3’UTR
rs7604810	chr2	239061627	KLHL30	Intergenic
rs4459687	chr2	239203368	ncRNA/PER2	Intronic SNV
rs1714416	chr3	40150543	MYRIP	Intronic SNV
rs7627014	chr3	40309316	EIF1B-AS1	Intronic SNV
			(Near MYRIP)	
rs908078	chr3	5024771	BHLHE40	Synonymous
rs2249436	chr3	5019764	BHLHE40	Intronic SNV
rs34870629	chr3	5025650	BHLHE40	3’UTR
rs34883305	chr3	5025645	BHLHE40	3’UTR
rs74439275	chr3	5025654	BHLHE40	3’UTR
rs2110585	chr5	133512621	SKP1	5’ UTR SNV
rs3815506	chr5	133509736	SKP1	Intronic SNV
rs73791514	chr5	133509752	SKP1	Intronic SNV
rs1058023	chr5	133483382	TCF7	3’ UTR
			(Near SKP1)	
rs243477	chr7	148456154	CUL1	Intronic SNV
rs10746964	chr9	77245494	RORB	Intronic SNV
rs7939846	chr11	13303337	ARNTL	Intronic SNV
				
rs4963957	chr12	26280533	SSPN	Intronic SNV
			(Near BHLHE41)	
rs61376834	chr12	56814656	TIMELESS	Missense Ile/Thr
rs605153	chr13	77569901	CLN5	Intronic SNV
			(Near FBXL3)	
rs5822477	chr17	80200398	CSNK1D	Deletion/Insertion
rs56408410	chr17	8052415	PER1	Intronic SNV
rs5757055	chr22	38740853	CSNK1E	Intronic SNV
rs35351192	chr22	38740868	CSNK1E	Insertion/Deletion

Abbreviations: CSNK1D: casein kinase 1 delta; SKP1: S-phase kinase associated protein 1; BHLHE40: basic helix-loop-helix family member e40; TCF7: transcription factor 7 (T-cell specific, HMG-box); NPAS2: neuronal PAS domain protein 2; ARNTL: aryl hydrocarbon receptor nuclear translocator like; MYRIP: myosin VIIA and Rab interacting protein; SSPN: sarcospan; EIF1B-AS1: EIF1B antisense RNA1; KLHL30: kelch like family member 30; CLN5: ceroid-lipofuscinosis, neuronal 5; FBXL3: FBOX leucine rich repeat protein 3; CUL1: cullin 1; RORB: RAR related orphan receptor B; PER1: period circadian clock 1; nc: non-coding; SNV: single nucleotide variation; UTR: untranslated region; PSQI: Pittsburgh Sleep Quality Index.

**Table 3:** Association of genetic variants with PSQI scores.

ID	Major/Minor Genotypes	MAF	PSQI Score		p-value

			(Log Mean ± SD)		

			Reference	Alternative	

rs5822477	TT/TTCTC	0.050	1.84 ± 0.57	0.96 ± 0.91	0.0015
rs2110585	CC/CA	0.058	1.84 ± 0.57	1.06 ± 0.90	0.0025
rs3815506	AA/AG	0.050	1.83 ± 0.57	1.01 ± 0.97	0.0028
rs73791514	AA/AT	0.050	1.83 ± 0.57	1.01 ± 0.97	0.0028
rs2249436	TT/TC	0.050	1.83 ± 0.59	1.01 ± 0.83	0.0029
rs1058023	CC/CT	0.092	1.86 ± 0.54	1.25 ± 0.92	0.0041
rs3841571	A/AG….GGGG	0.050	1.68 ± 0.64	2.38 ± 0.46	0.013
rs7939846	GG/GA	0.075	1.84 ± 0.64	1.26 ± 0.59	0.014
rs1714416	TT/TC	0.050	1.82 ± 0.61	1.15 ± 0.80	0.018
rs908078	TT/TC	0.108	1.65 ± 0.65	2.13 ± 0.58	0.018
rs4963957	TT/TC	0.075	1.83 ± 0.61	1.29 ± 0.76	0.022
rs5757055	CC/CG	0.158	1.62 ± 0.66	2.03 ± 0.57	0.023
rs7627014	AA/AT	0.058	1.68 ± 0.66	2.26 ± 0.31	0.028
rs1053095	TT/TA	0.217	1.91 ± 0.63	1.54 ± 0.64	0.030
rs7604810	GG/GA	0.142	1.87 ± 0.52	1.46 ± 0.88	0.030
rs61376834	AA/AG	0.075	1.67 ± 0.68	2.18 ± 0.32	0.033
rs605153	GG/GA	0.117	1.85 ± 0.53	1.44 ± 0.93	0.042
rs243477	CC/CT	0.058	1.81 ± 0.62	1.28 ± 0.79	0.042
rs35351192	ACAC/ACA	0.058	1.69 ± 0.67	2.22 ± 0.26	0.046
rs4459687	TT/TC	0.050	1.80 ± 0.63	1.32 ± 0.82	0.088
rs10746964	TT/TC	0.050	1.80 ± 0.60	1.32 ± 1.01	0.088
rs56408410	GG/GA	0.083	1.69 ± 0.62	2.07 ± 0.8	0.089
rs34870629	GG/GT	0.092	1.68 ± 0.69	2.05 ± 0.36	0.099
rs34883305	GG/GC	0.092	1.68 ± 0.69	2.05 ± 0.36	0.099
rs74439275	GG/GA	0.092	1.68 ± 0.69	2.05 ± 0.36	0.099

Abbreviations: MAF: minor allele frequency; PSQI: Pittsburgh Sleep Quality Index.

## Discussion

Studies have reported that 30–60% of breast cancer patients have poor sleep quality before receiving adjuvant chemotherapy and continue to have these symptoms even one year after the start of chemotherapy [34,35]. However, there is much variability in sleep quality symptoms among breast cancer patients, and it is not known why certain patients develop these symptoms and others do not.

While environmental factors influence sleep, a growing body of evidence suggests genetic modulation of sleep quality [36]. Its genetic regulation is substantiated by the identification of polymorphisms in specific sleep disorders and the existence of familial sleep disorders [15]. Twin studies have shown sleep heritability (h^2^) of 0.30–0.50. However, no study was located that evaluated the association of genetic variants in circadian pathway genes and sleep quality among breast cancer patients.

**Figure 2:**
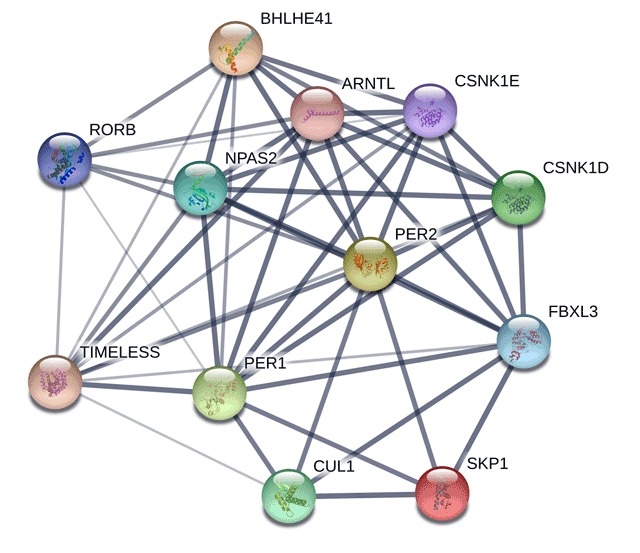
Known and Predicted Gene Networks. Identified genes with variants associated with PSQI were entered into String Version 10.5 to determine interacting gene networks. Line thickness indicates strength of data support.

Findings from this exploratory study suggest that circadian genes may play a role in sleep quality in women with breast cancer. Twenty-five genetic variants were associated with the global PSQI score. The genetic variants found were throughout the genome (chromosomes 2, 3, 5, 7, 9, 11, 12, 13, 22) and represented 15 genes including CSNK1D & E, SKP1, BHLHE40 & 41, NPAS2, ARNTL, MYRIP, KLHL30, TIMELESS, FBXL3, CUL1, PER1 & 2, and RORB. These genes are critical components of the circadian pathway that may play a role in sleep quality. The primary transcription/translation feedback loop of the pathway includes ARNTL, which forms a heterodimer complex with either CLOCK or NPAS2 and activates transcription of PERs (PER1, 2 & 3) and CRYs (CRY1 & 2) (Figure 2). Then PER and CRY form a negative feedback loop that represses their own transcription by acting on the heterodimer complex [37]. There is also evidence that TIMELESS is required for circadian regulation and interacts with CRY and PER proteins. The ARNTL heterodimers also induce another regulatory loop that activates RORA & B and subsequent transcription of ARNTL. Many other circadian proteins undergo post-translational modifications that affect the function of the feedback loops, including phosphorylation, acetylation, sumoylation and ubiquitination (CSNK1D & E, FBXL3, SKP1, CUL1).

Previous studies have investigated genetic markers of sleep in the general population using circadian candidate gene and genome wide association (GWA) study designs. The effects of PER3 variants, especially the variable number tandem repeats (VNTR), have been associated with many phenotypes including diurnal preference and sleep loss/circadian misalignment. In our study we did not find an association of the VNTR with sleep quality; however, this lack of replication may be due to the small size of the current study. In a candidate gene study, ARNTL (rs3816358) and NPAS2 (rs3768984) were associated with later actigraphic sleep and wake onset time in an elderly male population (n = 2,527) [38]. ARNTL was also found to be associated with sleep duration in a GWA study, though at a loci 40kb upstream of the gene, rs41348446 [39]. We also found associations between ARNTL and NPAS2 with sleep quality, however at different loci than previous studies.

Most variants found in this study are located in intronic and untranslated regions. The functional significance of these variants is unclear due to their possible linkage with other polymorphisms nearby. We found a missense variant in TIMELESS that was associated with poorer sleep quality as assessed by PSQI. While no studies have documented this association, the missense variant could potentially alter protein folding and interaction with PER and CRY, and thereby inhibit the primary transcription/translation loop in the circadian pathway, thus resulting in sleep disturbance.

Not only can circadian genes directly affect an individual’s susceptibility to sleep disturbances, studies have found that genetic variation in circadian genes are risk factors for breast cancer, most likely by impacting the biological pathways that regulate DNA damage and repair, carcinogen metabolism and or detoxification, cell-cycle progression and apoptosis. One of the first epidemiologic studies correlated PER3 variants with increased risk of breast cancer [3]. This circadian-cancer link was confirmed in a meta-analysis showing association between risk of cancer and variants in NPAS2, RORA, RORB, and CLOCK [40,41,42]. As this study included only women with breast cancer, the link between breast cancer risk and genetic variants could not be ascertained.

There are several strengths and limitations of this study. To our knowledge, it is the first to include an extensive selection of variants and genes in the circadian rhythm pathway in association with self-reported sleep in a sample of women with breast cancer. We included 5,279 genetic variants found in 26 circadian genes. We used statistical methods to identify the association between self-reported sleep quality and circadian-related genetic variants. However, findings from this study must be interpreted with caution due to the small sample size. Larger studies replicated in several populations are needed to fully understand the biological implications of circadian pathway genes and their role in sleep disturbance among breast cancer patients. Our results also indicate that the exome sequencing methodology detected not only coding polymorphisms in the genome, but also a significant number of non-coding variants. We have since modified our sequencing protocol to more precisely target exomes and will use this newer technology to increase the probability of detecting functional coding genetic variants in a larger study. Another limitation of this study is that we used sleep quality as a subjective measure. The PSQI was completed only at one time and timing varied among participants. Future studies could focus on patterns of sleep and sleep-wake activity rhythm using objective measures and/or a biomarker such as melatonin, and their association with circadian genes.

## Conclusions

Despite these limitations, findings from this study provide preliminary evidence for a role of circadian rhythm pathway genes in sleep quality among women with breast cancer. We conclude that these results merit further studies using larger sample sizes and more precise exome sequencing technology to allow for confirmatory analyses and identification of functional genetic variants, respectively. This research team is seeking funding to conduct a larger study in the near future.
